# Equivalence between Step Selection Functions and Biased Correlated Random Walks for Statistical Inference on Animal Movement

**DOI:** 10.1371/journal.pone.0122947

**Published:** 2015-04-21

**Authors:** Thierry Duchesne, Daniel Fortin, Louis-Paul Rivest

**Affiliations:** 1 Département de mathématiques et de statistique, Université Laval, Québec, Québec, Canada; 2 Département de biologie and Centre d'étude de la forêt, Département de Biologie, Université Laval, Québec, Québec, Canada; CNRS (National Center for Scientific Research), FRANCE

## Abstract

Animal movement has a fundamental impact on population and community structure and dynamics. Biased correlated random walks (BCRW) and step selection functions (SSF) are commonly used to study movements. Because no studies have contrasted the parameters and the statistical properties of their estimators for models constructed under these two Lagrangian approaches, it remains unclear whether or not they allow for similar inference. First, we used the Weak Law of Large Numbers to demonstrate that the log-likelihood function for estimating the parameters of BCRW models can be approximated by the log-likelihood of SSFs. Second, we illustrated the link between the two approaches by fitting BCRW with maximum likelihood and with SSF to simulated movement data in virtual environments and to the trajectory of bison (*Bison bison* L.) trails in natural landscapes. Using simulated and empirical data, we found that the parameters of a BCRW estimated directly from maximum likelihood and by fitting an SSF were remarkably similar. Movement analysis is increasingly used as a tool for understanding the influence of landscape properties on animal distribution. In the rapidly developing field of movement ecology, management and conservation biologists must decide which method they should implement to accurately assess the determinants of animal movement. We showed that BCRW and SSF can provide similar insights into the environmental features influencing animal movements. Both techniques have advantages. BCRW has already been extended to allow for multi-state modeling. Unlike BCRW, however, SSF can be estimated using most statistical packages, it can simultaneously evaluate habitat selection and movement biases, and can easily integrate a large number of movement taxes at multiple scales. SSF thus offers a simple, yet effective, statistical technique to identify movement taxis.

## Introduction

Animal movement is a fundamental mechanism shaping the structure and dynamics of populations, communities, and ecosystems [1]. Accordingly, movement analyses have been used to clarify the interactions within and among trophic levels. For example, the role of intra-guild interaction in structuring predator-prey interactions was revealed through the adjustment of large African carnivore movements with respect to prey distribution and the location of inter-specific competitors [2]. Also, Latombe et al. [3] showed that large herbivores momentarily adjust their movements to increase their use of food-rich areas following the recent passage of predators. These few studies follow the rapid development of movement ecology that has been observed in recent years [4], a development that is even expected to accelerate because of recent technological advances [5]. Radio-tracking technology is becoming available for increasingly smaller animal species, and at much lower prices. Nathan et al. [1] suggest that such technological developments are in part responsible for the shift from movement analysis being based on population redistribution (Eulerian approach) to individual path characteristics (Lagrangian approach). The authors further suggest that progress in movement ecology will entail revolutionary improvements in analytical techniques. We contend that, as a broader range of people become interested in movement analysis, rapid empirical advances will also require robust statistical techniques that can be easily implemented by non-specialists.

A large number of analytical techniques is available to evaluate internal and external factors shaping movement trajectories [6–9]. Two commonly used approaches, which are consistent with the Lagrangian approach, are the classic biased correlated random walk (BCRW) [7] and the more recent step selection function (SSF) [10]. A BCRW is a mathematical model that describes animal movement in terms of a small number of parameters [11], whereas SSF is a non parametric analysis technique that characterizes animal movement by determining how the sites visited by an animal differ from those available locally but not visited. The assessment of movement bias in preferred directions or towards specific targets [2,7,11] can be done from field data by either fitting a BCRW model or carrying out an SSF analysis. Both techniques are used to model and understand the determinants of animal movement from multiple taxa. For example, fitting a BCRW model was used to tease apart behavioural states that are displayed by grey seal (*Halichoerus grypus*) [12], to evaluate functional connectivity [13] and edge effects [14] for butterflies, to assess the influence of topography on the foraging behaviour of elk (*Cervus canadensis*) [15], and to evaluate theoretical expectations of spatial information on the success of foragers [16]. Likewise, SSFs have been used, for example, to study the response of elk and woodland caribou (*Rangifer tarandus caribou*) to wolf (*Canis lupus*) distribution [3,10], together with the reactions of wolf [17], grizzly bear (*Ursus arctos horribilis*) [18], snowshoe hare (*Lepus americanus*) [19], roe deer (*Capreolus capreolus*) [20] and multiple bird species [21] to various landscape features. Fitting a BCRW model and carrying out an SSF analysis thus can both characterize various movement taxes. What remains unclear, however, is the extent to which these two techniques can lead to similar inferences. Whereas the former directly models an animal’s movement (bearing and distance) through a stochastic process, the latter models its probability of selecting a location among available ones.

Here we provide a mathematical proof that these two movement analysis approaches are equivalent under a broad set of conditions. First, we introduce two BCRW models and discuss the estimation of their parameters with the maximum likelihood method. Second, we consider an SSF analysis featuring angular components for both a directional persistence and a directional bias, and show that the resulting SSF estimates are mathematically equivalent to BCRW maximum likelihood estimates. This theoretical equivalence is then confirmed empirically through a simulation study and a field study on the directionality of trails that were made by bison (*Bison bison* L.). We focused on the estimation of directional biases because the orientation of steps with respect to habitat features is one of the most fundamental aspects of BCRW [7,15,22], and it is generally overlooked in SSF studies [9,10].

### Specification of biased correlated random walks

BCRW is considered as a highly flexible and powerful discrete-step model of single-scale single mode animal movements, as it combines long and short term attractiveness with various degrees of stochasticity [23]. The long-term attractiveness can, for instance, be associated to a patch that is rich in nutrients while directional persistence accounts for short-term effect (i.e., directional persistence implies that the expected direction at a given point in time is that of the previous move bearing) [14]. When implementing a BCRW, the path of the animal is discretized and the movement between times *t* and *t+1* is a 2D step vector, for *t* = 0,…,*T*. It is convenient to use a polar representation of this vector, (*y*_*t*_, *d*_*t*_) where the step angle *y*_*t*_ ∈ [−*π*, *π*) gives the direction of the movement with respect to a reference direction, e.g. north, and *d*_*t*_ is the step length.

In a BCRW, let *ψ*_*t*_ be an angle giving the direction toward the target (i.e., the long-term attractor) at time *t*; this is the angle, with respect to the reference direction, of a line joining the animal’s position at time *t* and the target’s position. The step angle *y*_*t*_ is the result of a compromise between directional persistence, i.e., the innate tendency to move in the same direction as the previous step [7], and movement in response to the target in direction *ψ*_*t*_. The compromise nature of the random step angle *y*_*t*_ can be modeled by setting its mean direction *μ*_*t*_ to the vector direction of

$$v_{t}=\left(\begin{array}{c} \text{cos}(y_{t-1})\\ \text{sin}(y_{t-1}) \end{array}\right)+\beta \left(\begin{array}{c} \text{cos}(\psi_{t})\\ \text{sin}(\psi_{t}) \end{array}\right).$$ 1

Thus, *μ*_*t*_ = atan{sin(*y*_*t*−1_) + *β* sin(*ψ*_*t*_), cos(*y*_*t*−1_) + *β* cos(*ψ*_*t*_)}, where atan(*a*,*b*) = atan (*a/b*) for *b*>0 and atan *(a/b)* + *π* for *b*<0 [24,25]. Many ecological papers, e.g. [12,14,15,22], sets the mean direction *μ*_*t*_ equal to a convex combination, *μ*_*t*_ = *λy*_*t*−1_ + (1−*λ*)*ψ*_*t*_ for some *λ* in (0,1). As McClintock et al. (2012) pointed out, this is not satisfactory, especially if |*y*_*t*−1_−*ψ*_*t*_| > *π*, because adding, or substracting, *2 π* to either *y*_*t-1*_ or *ψ*_*t*_ leads to different numerical values for *μ*_*t*_. Using Eq 1 gives a more stable definition, invariant to the addition or the subtraction of *2π* to either angle. It can therefore be easily extended to more than one directional bias as illustrated below.

Parameter *β* weights the attractive strength of the target relative to directional persistence in the compromise that the animal makes when it chooses the movement angle for its next step. When *β* = 0, *μ*_*t*_ = *y*_*t-1*_ and the model reduces to a correlated random walk, whereas when *β* goes to infinity *μ*_*t*_ = *ψ*_*t*−1_ and we get a biased random walk (Benhamou, 2014). Overall we demonstrate that the expected movement direction in BCRW should be defined using Eq 1, because it gives a more stable definition than one that uses a convex combination of *y*_*t-1*_ and *ψ*_*t*_.

### Estimation of the parameter *β* of biased correlated random walk

Given some data {(*y*_*t*_, *ψ*_*t*_): *t* = 0,…, *T*}, we consider two circular regression models to estimate the parameter *β* of the BCWR, viz., the angular model and the consensus model.

The *angular model* is obtained by assuming that the step angles *y*_*t*_ have a von Mises distribution, with a mean direction *μ*_*t*_ and a concentration parameter *κ* > 0. The corresponding density is

$$f_{vMa}(y_{t})=\frac{1}{2\pi I_{0}(\kappa )}\text{exp}\left\{\kappa \;\text{cos}(y_{t}-\mu_{t})\right\},\;y_{t}\in [-\pi ,\pi )\;,$$ 2

where *I*_*0*_ denotes a modified Bessel function [26]. The angular estimation method consists of estimating the parameters (*β*, *κ*) by maximizing the likelihood that is constructed with Eq 2 [24,25].

The *consensus model* assumes that the error concentration parameter depends upon the degree of agreement between the two possible goals of the animal, i.e., moving forward versus going towards a target. The agreement is measured by the length $\ell_{t}$ of vector **v**_*t*_ in Eq 1, $\ell_{t}={\left[{\left\{\text{sin}(y_{t-1})+\beta \text{sin}(\psi_{t}\;)\right\}}^{2}+{\left\{\text{cos}(y_{t-1})+\beta \text{cos}(\psi_{t}\;)\right\}}^{2}\right]}^{1/2}$. When *β* is positive, maximum length, $\ell_{t}=1+\beta$, occurs when directional persistence leads directly to the target, i.e., *ψ*_*t*_ = *y*_*t*−1_, whereas minimum length, $\ell_{t}=|1-\beta |$, is obtained when directional persistence pushes the animal in a direction opposite to that of the target, i.e., *ψ*_*t*_ = *y*_*t*−1_ +*π*. As an alternative to Eq 2, the consensus model assumes that the step angles *y*_*t*_ have a von Mises distribution with a mean direction *μ*_*t*_ and a concentration parameter $\kappa \ell_{t}$ for some κ > 0. This simultaneous modeling of the mean direction and the concentration parameter has been advocated by Presnell et al. [27], in particular due to the ease with which the likelihood can be numerically maximized. Given that $\text{sin}\left(\mu_{t}\right)=\left\{\text{sin}\left(y_{t-1}\right)+\beta \;\text{sin}\left(\psi_{t}\right)\right\}/\ell_{t}$ and $\text{cos}\left(\mu_{t}\right)=\left\{\text{cos}\left(y_{t-1}\right)+\beta \;\text{cos}\left(\psi_{t}\right)\right\}/\ell_{t}$, the von Mises density for the consensus model simplifies to:

$$f_{vMc}(y_{t})=\frac{1}{2\pi I_{0}(\kappa_{1}\ell_{t})}\text{exp}\left\{\kappa_{1}\;\text{cos}(y_{t}-y_{t-1})+\kappa_{2}\;\text{cos}(y_{t}-\psi_{t})\right\},\;y_{t}\in [-\pi ,\pi )\;,$$ 3

where *κ*_1_ = *κ* and *κ*_2_ = *κβ*. This density belongs to the exponential family and the estimation of (*κ*_1_, *κ*_2_), and of *β* = *κ*_2_/*κ*_1_ is easily carried out by maximum likelihood. This defines the consensus estimation method. In summary, the parameter *β* in a BCRW can be directly estimated by maximum likelihood by maximizing the likelihood of one of the two models (Eq 2 or Eq 3), respectively the angular and consensus models.

### Step selection function for estimating the parameter *β* of biased correlated random walk

We now provide a third method that, based on step selection function (SSF) [10], can be used to estimate the parameter *β* of Eq 1. SSF uses, for each time point *t*, *J* control step angles $\delta_{t}^{\left(j\right)}$, *j = 1*,*…*,*J* that are randomly selected among the *T* step angles {*y*_*t*_} in the data set. The SSF for the BCRW of Eq 1 has two explanatory variables, the cosines of the turning angle *y*_*t—*_ *y*_*t-1*_ and of the angle to the target *y*_*t*_−*ψ*_*t*_. Its likelihood, which is derived in section 8.4 of [28], is proportional to

$$L_{a}(\kappa_{1},\kappa_{2})\approx \prod_{t=1}^{T}\frac{J\;\text{exp}\left\{\kappa_{1}\;\text{cos}(y_{t}-y_{t-1})+\kappa_{2}\;\text{cos}(y_{t}-\psi_{t})\right\}}{\text{exp}\left\{\kappa_{1}\;\text{cos}(y_{t}-y_{t-1})+\kappa_{2}\;\text{cos}(y_{t}-\psi_{t})\right\}+\sum_{j=1}^{J}\text{exp}\left\{\kappa_{1}\;\text{cos}(\delta_{t}^{\left(j\right)}-y_{t-1})+\kappa_{2}\;\text{cos}(\delta_{t}^{\left(j\right)}-\psi_{t})\right\}}.$$ 4

The SSF estimation method consists of maximizing *L*_*a*_(*κ*_1,_ *κ*_2_) to estimate *κ*_1,_ *κ*_2_, and thus, *β* = *κ*_2_/*κ*_1_. We now show that this estimation method is related to the consensus estimation method.

Suppose that the control step angles $\delta_{t}^{\left(j\right)}$ are uniformly distributed over (0, 2 *π*). The denominator of *L*_*a*_(*κ*_1_, *κ*_2_) for time *t* is equal to

$$\frac{1}{J}\left[\text{exp}\left\{\kappa_{1}\;\text{cos}(y_{t}-y_{t-1})+\kappa_{2}\;\text{cos}(y_{t}-\psi_{t})\right\}+\sum_{j=1}^{J}\text{exp}\left\{\kappa_{1}\;\text{cos}(\delta_{t}^{\left(j\right)}-y_{t-1})+\kappa_{2}\;\text{cos}(\delta_{t}^{\left(j\right)}-\psi_{t})\right\}\right].$$

When *J* is large, the term involving *y*_*t*_ is negligible and the denominator at time step *t* is

$$\frac{1}{J}\sum_{j=1}^{J}\text{exp}\left\{\kappa_{1}\;\text{cos}(\delta_{t}^{\left(j\right)}-y_{t-1})+\kappa_{2}\;\text{cos}(\delta_{t}^{\left(j\right)}-\psi_{t})\right\}\approx I_{0}(\kappa \ell_{t}).$$

This equation derives from the Law of Large Numbers [29]: as *J* goes to ∞, the sample average of $\left\{\text{exp}\left[\kappa_{1}\;\text{cos}(\delta_{t}^{\left(j\right)}-y_{t-1})+\kappa_{2}\;\text{cos}(\delta_{t}^{\left(j\right)}-\psi_{t})\right]:j=1,\ldots ,J\right\}$ converges to the expectation $E\left[\exp\left\{\kappa_{1}\;\cos(\delta_{t}^{\left(1\right)}-y_{t-1})+\kappa_{2}\;\text{cos}(\delta_{t}^{\left(1\right)}-\psi_{t})\right\}\right]=\frac{1}{2\pi }\int_{\pi }^{-\pi }\text{exp}\left\{\kappa_{1}\;\text{cos}(\theta -y_{t-1})+\kappa_{2}\;\text{cos}(\theta -\psi_{t})\right\}d\theta =I_{0}(\ell_{t}\kappa )$.

The SSF likelihood that is given in Eq 4 is approximately equal to:

$$L_{a}(\kappa_{1},\kappa_{2})\approx \prod_{t=1}^{T}\text{exp}\left\{\kappa_{1}\;\text{cos}(y_{t}-y_{t-1})+\kappa_{2}\;\text{cos}(y_{t}-\psi_{t})\right\}/I_{0}(\kappa_{1}\ell_{t}).$$

This is the likelihood function for the consensus model of Eq 3. Thus, the empirical SSF estimates for *κ*_1_, *κ*_2_, and *β* = *κ*_2_/*κ*_1_ should be close to those that are obtained by the consensus estimation method, as long as the control angles have a uniform distribution; this property is investigated in the Monte Carlo study that is presented in the next section.

### Monte Carlo study comparing *β* estimators

Three methods are available to estimate the parameter *β*, which quantifies the importance of the target on animal movement relative to directional persistence. They are: (i) the angular estimation method, which uses the likelihood that is constructed using Eq 2; (ii) the consensus estimation method, which uses the likelihood that is constructed using Eq 3; and (iii) the SSF estimation method, with cos(*y*_*t—*_ *y*_*t-1*_) and cos(*y*_*t*_−*ψ*_*t*_) as explanatory variables, see Eq 4. We used Monte Carlo simulations to compare the *β* values that were provided by the three estimation methods.

To avoid multicollinearity problems that arise when simulating data for a single animal, all simulations involved two animals moving independently on a 2D simulated landscape of 1024 x 1024 pixels. The simulated landscape was developed following [9]. Directional bias was towards the center pixel of the landscape. The starting locations of the two animals were randomly sampled, one in the northwest corner of the landscape map and the other in the southeast corner. Each animal was observed for 61 steps, yielding 120 observations contributing to the likelihood of each estimation method. This approach ensured that the series of data were long enough to make the model parameters estimable, yet not long to the point where the animals would spend the latter part of the series meandering about their long term target.

Three sets of simulations were carried out, each with 500 repetitions. The first simulation model used a map that was comprised of three habitat types occupying respectively 80%, 10%, and 10% of the simulated landscape (see S1 Appendix). The map was constructed by discretizing the realization of a Gaussian random spatial process (details available in S1 Appendix). An animal’s displacement at time *t* was simulated using a discrete choice model, which enables us to assess the robustness of the proposed methods, i.e., to verify whether they are able to identify the directional biases in situations where the noise is not exactly as modeled in Eq 2 and 3. First, among the pixels that were more than 60 pixels away from the animal’s current location, the closest pixels of habitats 2 and 3 are identified as candidates for the next step. One pixel is selected randomly among the candidate pixels; the probability of selecting pixel *k* among these candidate pixels is proportional to $\text{exp}\{\kappa_{1}\;\text{cos}(y_{t}^{\left(j\right)}-y_{t-1})+\kappa_{2}\;\text{cos}(y_{t}^{\left(j\right)}-\psi_{t})\}$, where $y_{t}^{\left(j\right)}$ is the angle of the segment joining the animal’s position at time *t* to candidate pixel *k*, whereas *ψ*_*t*_ is the angle of the segment joining the animal’s position at time *t* to the long term target. Thus, pixels in the direction of the previous displacement or in the direction of the target are assigned higher probabilities. Three sets of values for (*κ*_1_, *κ*_2_) were used in the simulations, viz., (2, 2), (2, 0.5) and (0.5, 0.5). The pair (*κ*_1_, *κ*_2_) = (0.5, 2) was found to be uninteresting since the animals reached the target in a few steps and spent most of their time meandering around.

The animals’ displacement rules in the second and third simulation models did not depend upon the type of habitat and all steps had a length of *d*_*t*_ = 60 units. In the second set of simulations, the movement direction, *y*_*t*_, was simulated using the consensus model of Eq 3, with *κ* = *κ*_1_ and *β* = *κ*_2_/*κ*_1_. For the third simulation model, *y*_*t*_ was simulated according to the angular regression model of Eq 2, with *β* = *κ*_2_/*κ*_1_ and von Mises errors with respective concentration parameters 3, 2, and 0.75 for the three (*κ*_1_, *κ*_2_) pairs, i.e., (2, 2), (2, 0.5) and (0.5, 0.5).

The SSF estimation method involves a comparison between observed step angles, *y*_*t*_, with control step angles $\delta_{t}^{\left(j\right)}$ as in Eq 4. The likelihood *L*_*a*_(*κ*_1_, *κ*_2_) in (4) was calculated using, for each *t*, *J* = 10 random step angles, which were randomly sampled among the 120 observed step angles for that simulation. We used Kuiper’s test of uniformity [26] to verify whether or not the distribution of the observed *y*_*t*_’s from which we sampled the control step angles was close to the uniform distribution for each of the 9 simulation scenarios. Except in the case of the angular models with (*κ*_1_, *κ*_2_) = (2, 0.5) and (*κ*_1_, *κ*_2_) = (0.5, 0.5) that yielded *P* < 0.10, all tests had *P* > 0.15, indicating no significant differences between the empirical and uniform distributions. The conditions for the asymptotic equivalence of the SSF estimators and those that were obtained from the consensus model were approximately met.

### Simulation results

**Fig 1:**
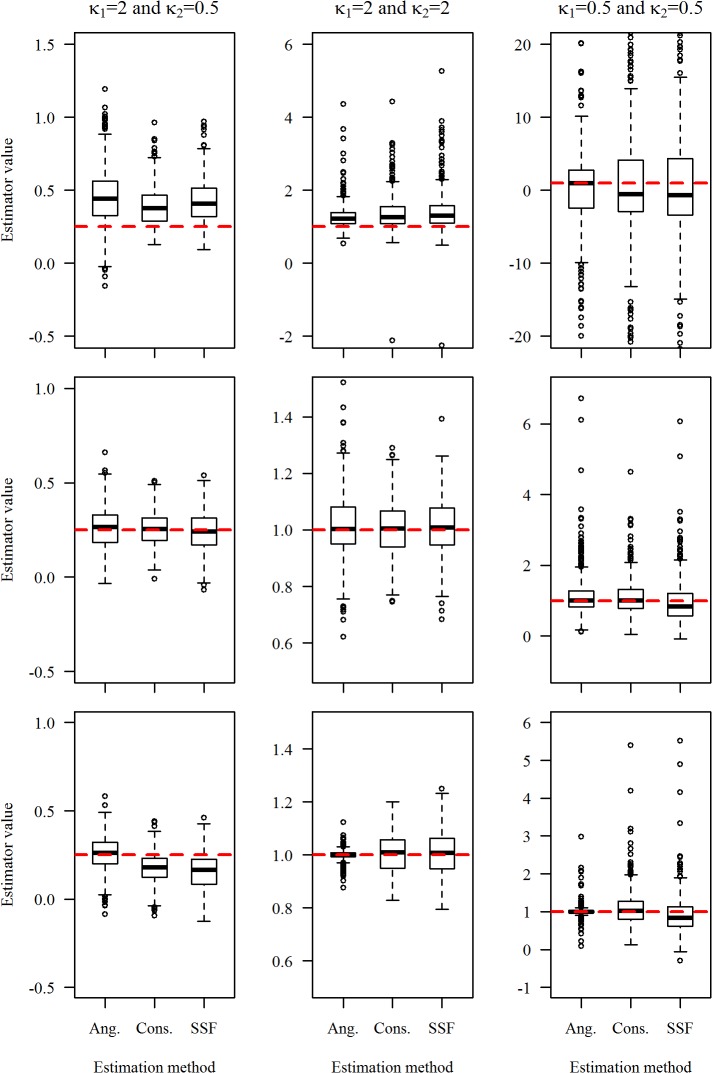
Comparison of the three estimators of *β* = *κ*_*2*_/*κ*_*1*_ for 9 simulation scenarios. Each boxplot summarizes values of the estimates of parameters β = κ_2_/κ_1_ that were obtained with the angular (Ang.), consensus (Cons.) and empirical SSF (SSF) estimation methods from 500 simulations under the discrete choice model (row 1), the consensus simulation model (row 2), and the angular simulation model (row 3). The red horizontal dashed line indicates the true parameter value.

Fig 1 compares the three estimators of *β* = *κ*_2_/*κ*_1_ for the 9 simulation scenarios. All three methods estimate *β* well (Fig 1). The consensus and SSF estimators are very similar. The random control locations entering into the SSF likelihood, however, make the SSF estimator slightly more variable than the consensus estimator. For the discrete choice simulation model, the angular estimation method exhibits a small bias when *κ*_1_ = 2, *κ_2_* = 0.5. It is superior to some degree, however, to the other two when *κ*_1_ = 0.5, *κ_2_* = 0.5, i.e., when the effect of directional persistence and the target’s attraction are small. For the consensus simulation model, the three methods are virtually equivalent, with a small advantage to the consensus estimator as it is the maximum likelihood estimator under the simulation model. For the angular simulation model, the consensus estimator is slightly better than the SSF estimator. The angular estimator, which is the maximum likelihood estimator under the simulation model, is somewhat better than the other two.

## Orientation of Bison Trails with Respect to Landscape Features

### Ethics Statement

The research was conducted in Prince Albert National Park, a Canadian National Park, in accordance with the research permit PA-2010–4552 provided by Parks Canada. Given that our study only relies on bison trails, data were collected without any manipulation of animals.

### Field data

We now estimate movement taxis based on each of the three methods, which were applied to bison trail data collected in Prince Albert National Park, Saskatchewan, Canada. The park is dominated by forest, with a bison trail network linking the discrete meadows that are found on the forest mosaic. Because we used the field data of Dancose et al. [30], with the addition of 22 bison trails, we will only summarize their method. Bison trails were systematically located by walking around the edges of meadows. Trails were then followed using a handheld Global Positional System unit until they reached another meadow. The information was then transferred to a Geographic Information System with a 10-m resolution. The trails were transformed into a string of successive pixels (10 × 10 m).

### Analysis

In the SSF analysis reported in their Table 5, Dancose et al. [30] investigate the relative importance of directional persistence, the direction of the next meadow, and the direction of the next canopy gap in the determination of a bison trail’s trajectory. The “time” variable is the position along the trail at time *t*, as if the observer were traveling on the trail towards the target meadow [30]. Given the position at time *t*, the position at time *t*+1 could in principle be one of the 8 pixels that neighbor the location occupied at time *t*. But the pixel that is occupied at time *t-1* is excluded so that there are 7 pixels that can be used at time *t+1*; one of the 7 gives the observed movement and the other 6 are used as controls in the SSF analysis [30]. The 218 trails generated a data set containing 5696 pixels and their controls.

The approach used to determine the link between animal movement and habitat characteristics often entails the development of candidate movement models that account for various combinations of habitat features that could influence movement decisions. The candidate models are then compared based on their relative empirical support. Given our purpose of contrasting the three methods, our analysis simply considered two movement taxes, together with directional persistence. For the consensus model, the generalization of Eq 3 to include two movement taxes is

$$f_{vMc}(y_{t})=\frac{1}{2\pi I_{0}(\kappa \ell_{t})}\text{exp}\left\{\kappa_{1}\;\text{cos}(y_{t}-y_{t-1})+\kappa_{2}\;\text{cos}(y_{t}-\psi_{1t})+\kappa_{3}\;\text{cos}(y_{t}-\psi_{2t})\right\},\;y_{t}\in [-\pi ,\pi )\;,$$

where *ψ*_1*t*_ and *ψ*_2*t*_ are the angles of the directions to the target meadow (TM) and to the closest canopy gap (CG) at time *t*. A generalization of the angular model (Eq 2) to two directional biases is constructed in a similar way. Estimates of the parameters *κ*_1_, *κ*_2_, and *κ*_3_ and *β*_1_ = *κ*_2_/*κ*_1_ and *β*_2_ = *κ*_3_/*κ*_1_ that were obtained with three estimation methods, whereas the robust standard errors that were calculated using sandwich estimates of the variance covariance matrix, which is valid regardless of the true error structure [31], are provided for the models that are defined by Eq 2 and 3. For the SSF estimates, the standard errors were obtained from the conditional logistic regression output. Finally, the standard errors of the *β* parameters for the SSF and the consensus estimates were calculated using the multivariate delta method (see S2 Appendix and S1 and S2 R codes).

## Results

The three sets of *β* estimates are remarkably similar (Table 1), even if the *κ* estimates for the SSF and the consensus model differ. The lack of differences might be due to the small number (6) of controls that were used in this analysis; estimation of the κs is discussed further in S1 Appendix. The SSF standard errors of the *β* parameters are slightly larger than those estimated by the other two methods; this was expected, given that only 6 controls were available for the SSF analysis. These numerical results differ slightly from those reported in Dancose et al (2011), because their data set included fewer bison trails. In addition, their SSF analysis included explanatory variables on the nature of the pixel being selected (i.e., land cover types), together with the directional information.

**Table 1:** Directional analysis of bison trails with respect to directional persistence (Direction. P.), the target meadow (TM), and the nearest canopy gap (CG).

Estimation Method	SSF		Consensus		Angular
Parameter	*κ*	*β*	*κ*	*β*	*β*
Direction. P.	10.21 (0.229)	NA	23.134 (0.456)	NA	NA
Bias to TM	2.410 (0.185)	0.236 (0.013)	5.156 (0.184)	0.223 (0.012)	0.210 (0.011)
Bias to CG	0.691 (0.07)	0.068 (0.007)	1.447 (0.103)	0.063 (0.005)	0.061 (0.005)

Three sets of estimates, using the step selection function (SSF) conditional logistic regression, the consensus and the angular circular regression models are provided, together with robust standard errors in parentheses.

## Discussion

Our study demonstrates that BCRW and SSF can lead to similar inference on the determinants of animal movement, under a broad set of conditions. We provide a mathematical proof that, when step angles are generated according to a von Mises concensus BCRW model, maximum likelihood estimation of the parameters of this BCRW model is equivalent to estimating these parameters by fitting an SSF with suitably chosen covariates and a large number of control step angles that are sampled from a uniform distribution. Further, our simulations and evaluation of factors influencing the spatial configuration of bison trails also demonstrate that inferences about the relative contribution (the *β* parameters) of movement taxis and directional persistence on inter-patch trajectories are virtually identical with both approaches. Thus, both BCRW and SSF are useful tools for identifying the factors controlling movement decisions.

In addition to the formal mathematical demonstration of the close link between SSF and BCRW, our Monte Carlo and empirical studies demonstrate the close agreement between the *β* estimates that were derived from the SSF and the two circular regression models that were considered. The equivalence between BCRW and SSF arises from the fact that both methods reveal the relative impact of multiple (generally two) factors responsible for directional biases in the mean direction of the animal’s movement at each step. The relative weight of multiple movement biases is not only relevant to anticipate movement paths given the pattern of landscape heterogeneity, but also to identify how the animal deals with conflicting objectives under various conditions [14,15,32]. For example, we showed that the orientation of bison trails reflects a combination of movement bias and taxis towards a canopy gap and the target meadow. Given the similarity between SSF and BCRW, we can wonder which approach should be used. Both methods are versatile and can be used to evaluate the role of external factors and internal states on movement decisions. Whereas BCRW is a stochastic model that lends itself well to simulation of animal movement in a neutral environment [23], SSF can be viewed a discrete choice model that can be used to simulate site selection from locally available sites [33].

SSFs are estimated from the comparison of observed steps paired with traveled ones. Generally, unused steps are drawn from the empirical distributions of turning angles and step lengths [10]. Forester et al. [9] showed that step length should be included in the SSF as a covariate when it is transformed with a linear spline function of distance to avoid potential bias. Here, we show that drawing control step angles from a uniform distribution yields models in the family of BCRW. In this context, SSFs could be developed by drawing step angles from a uniform distribution (this is similar to Forester et al.'s [9] proposal of drawing control locations uniformly in a disk around the previous location), and then including a term (e.g., cosine of the turning angle *y*_*t—*_ *y*_*t-1*_) that would account for the animal’s propensity for moving forward.

Whereas Benhamou [23] suggests that BCRW is the most flexible and discrete-step model of single analysis, we demonstrate that this type of analysis can be implemented within the SSF framework. A strong advantage of SSFs is that the models are estimated using conditional logistic regression, which is available in most statistical packages. Fitting a BCRW directly is usually more challenging because it requires more advanced programming skills, but codes are becoming increasingly available, e.g. [25]. SSFs can also include a combination of multiple movement biases, together with discrete choices among the different land cover types that can be traveled during the relocation time interval [30,34]. This addition might reflect the fact that animals may have multiple objectives acting at different spatio-temporal scales, such as the use of stepping stones while moving towards a distant target. The assessment of the interplay between multi-scale landscape structure and movement paths is an important objective of movement ecology [23]. Further, all of these determinants of movement decisions can be easily adjusted for other factors (e.g., group size, energy level) through the inclusion of interaction terms in the conditional logistic regression, see [35]. There are currently no straightforward ways of adding components for the selection of land type cover to a BCRW.

In contrast, BCRW has already been extended to allow for multi-state hidden Markov modeling [25]; additional methodological developments would be needed for implementing multi-state SSFs. Also, since the SSF approximates the BCRW normalizing constant with control locations, it yields *β* estimators with relatively larger variances. This issue can be circumvented, however, by increasing the number of control locations.

In conclusion, by reuniting BCRW and SSF, our study demonstrates that these two Lagrangian methods for movement analysis can both provide similarly robust insights into the mechanisms of animal distribution. These statistical techniques could provide parameters to conduct mechanistic home-range analysis based on advection-diffusion models by slightly modifying Moorcroft and Barnett’s [36] approach. Indeed, the odds of making a particular step under particular local habitat conditions could be directly extracted from SSF. They can also be important steps to implement Nathan et al.’s [1] theoretical framework for movement ecology, which is based on the internal state, motion, and navigation capacities of the individual and the external factors affecting its movements. We agree with Fagan et al. [37], who suggest in their review that SSF is a particularly promising tool for studying the role of memory in movement decisions. SSF could be used, for example, to assess whether or not individuals selectively move towards previously visited sites in a way that reduces uncertainty about future energy gains. Furthermore, SSF can be estimated with most statistical packages, making the method readily available to those interested in movement ecology and its applications. Because of its simplicity and versatility in the number and type of covariates that can be considered simultaneously, we contend that the use of SSF should accelerate the acquisition of knowledge on the determinants of animal movement and distribution.

## Supporting Information

S1 Appendix Landscape map and additional results for the simulation study. (DOCX)

S2 AppendixEstimation of the standard errors.Estimation of the standard errors of ${\hat{\beta }}_{1}$ and ${\hat{\beta }}_{2}$ using the variance covariance matrix of the $\hat{\kappa }$’s.(DOCX)

S1 Rcode Homogeneous or consensus model. R code required to replicate the simulation study when the data are simulated either from the homogeneous or consensus model.
(R) Click here for additional data file.

S2 Rcode Discrete choice model. R code required to replicate the simulation study when the data are simulated from the discrete choice model.
(R) Click here for additional data file.
