# Supplementary material for: Equivalence between Step Selection Functions and Biased Correlated Random Walks for Statistical Inference on Animal Movement
Source: PLoS One. 2015 Apr 21;10(4):e0122947. doi: 10.1371/journal.pone.0122947 (PMC4405542; doi:10.1371/journal.pone.0122947)
Supplement: S1 Appendix — (DOCX) [file pone.0122947.s001.docx]

**Equivalence between step selection functions and biased correlated random walks for statistical inference on animal movement. *PLoS ONE.***

S1 Appendix. *Landscape map and additional results for the simulation study*

***Landscape map***

The map that was used in the simulation study is shown in Fig. S1. To construct this map, we followed Forester et al. (2009) and generated a Gaussian random field over a grid of 1024 × 1024 pixels. We used the function GaussRF from the R package RandomFields (Schlater et al. 2013) to generate a Gaussian random field with an exponential covariance function, mean 0, variance 1, no nugget effect, and a scale of 21. We then defined habitat type 1 as all pixels for which the value of the simulated Gaussian variable was between its 10^th^ and 90^th^ percentiles, habitat type 2 as all pixels for which the value of the Gaussian variable was less than its 10^th^ percentile, and habitat type 3 as all other pixels. An example of the R code is as follows:

library(RandomFields)

model<-"exponential" # exponential covariance function

mean<-0

variance<-1

nugget<-0

scale2<-21

x<-1:1024

y<-1:1024

###Gaussian random field (GRF) based on an exponential covariance function

Map2 <- GaussRF(x=x, y=y, model=model, grid=TRUE,param=c(mean, variance, nugget, scale2))

Map2.ter <- quantile(c(Map2),probs=c(0.1,0.9))

Map2.patch <- 2*(Map2<Map2.ter[1])+1*(Map2>=Map2.ter[1])*(Map2<Map2.ter[2])+

3*(Map2>=Map2.ter[2])

image(x,y,Map2.patch,col=terrain.colors(3))

***Additional results for the simulation study***

Both the consensus and SSF estimators predict lower parameter estimates than the true $\kappa$ values (Fig. S2). The parameters of the discrete choice probability kernel governing an animal’s displacement cannot be recovered using these estimation methods. Only the relative importance $\beta=\kappa_{2}/\kappa_{1}$ of one direction, say directional persistence, with respect to another, is well estimated for the target (see Figure 1 in the main text). This is not the case when the data are simulated using the consensus model of Eq. 3. Fig. S3 shows that the two parameters are well estimated in these two cases.

Further simulations were carried out to understand why, as shown in Fig. S2, the parameters *κ*_1_ and *κ*_2_ were not well estimated when the simulations were carried out using a discrete choice model. The additional simulations compared three methods for selecting the controls for the conditional logistic regression analysis. The first one, which is referred to as “the gold standard,” assumes that the animal’s decision process is known: the controls are the pixels that received a non-null selection probability in the animal’s discrete choice selection for the displacement at a given step. The second one, which is referred to as “the doughnut,” is an intermediate method between the gold standard and the SSF. It uses a set of candidate pixels that is larger than the gold standard; the parcels in this set are all of those belonging to habitat 2 or 3 that are at a distance between 55 and 65 pixels from the current location. The third one is the standard SSF that selects control step angles from the empirical distribution of step angles. As the SSF in Eq. 4 does not involve step length, none are selected. Thus, this third method of selecting the controls does not ensure that control positions are in habitat 2 or 3. We found that the selection of the controls is the key to obtaining accurate estimators of $\kappa_{1}$ and $\kappa_{2}$ (Fig. S4). The gold standard estimators of $\kappa_{1}$, $\kappa_{2}$ and $\beta$ perform systematically better that the other two. The doughnut selection, which is closer to the true animal’s selection process, gives better estimators than the standard empirical SSF selection.

Literature cited

Forester J.D., Im H.K. & Rathouz P.J. (2009). Acccounting for animal movement in estimation of Resource Selection Functions: Sampling and data analysis. Ecology, 90, 3554-3565

Martin Schlather, Peter Menck, Richard Singleton, Ben Pfaff and R Core team (2013). RandomFields: Simulation and Analysis of Random Fields. R package version 2.0.66. <http://CRAN.R-project.org/package=RandomFields>


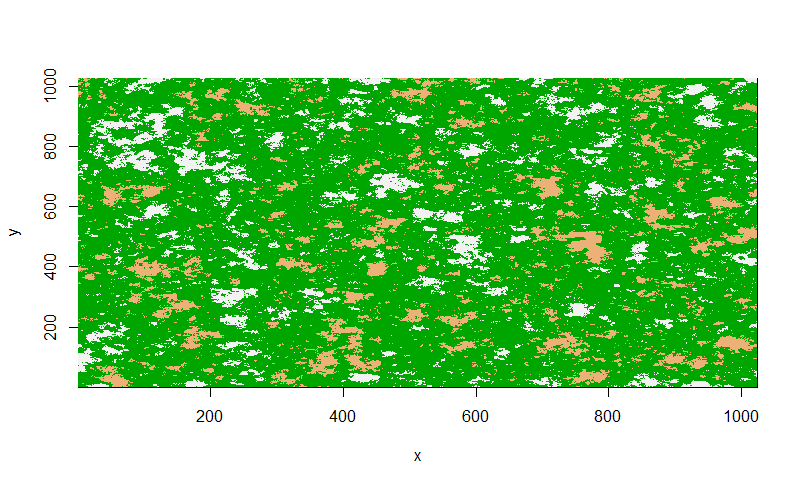
 Fig. S1. Map used in the simulation study. Habitat type 1 is comprised of all green pixels, whereas habitat types 2 and 3 correspond to the white and brown pixels, respectively.

**
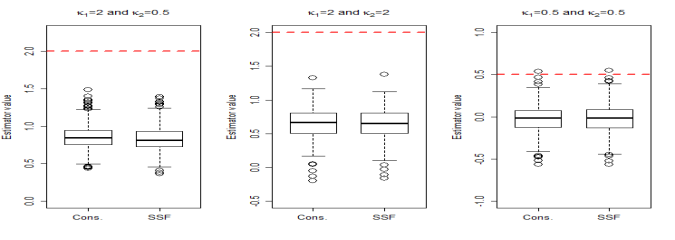
**

**
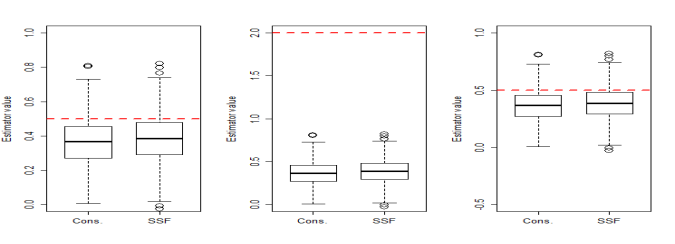
**

Fig. S2. Each boxplot summarizes the values of the estimates of parameters *κ*_1_ (row 1) and *κ*_2_ (row 2) that were obtained with the consensus (“Cons.”) and empirical SSF (“SSF”) methods from 500 simulations under the discrete choice simulation model. The red horizontal dashed line indicates the true parameter value.

**
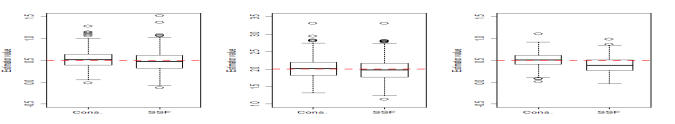

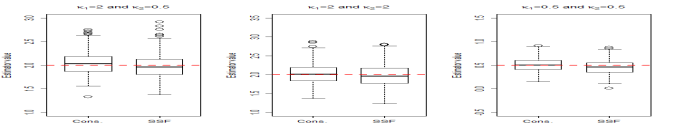
** Fig. S3. Each boxplot summarizes the values of the estimates of parameters *κ*_1_ (row 1) and *κ*_2_ (row 2) that were obtained with the consensus (“Cons.”) and empirical SSF (“SSF”) methods from 500 simulations under the consensus simulation model. The red horizontal dashed line indicates the true parameter value.

**
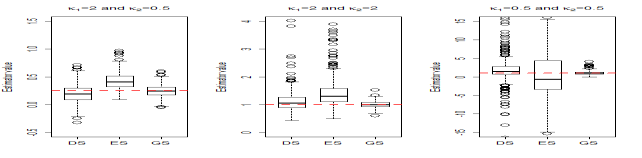
**
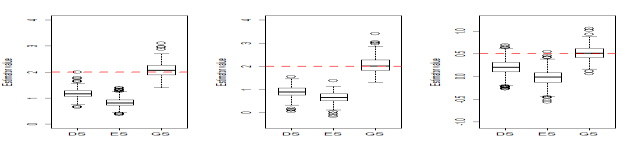

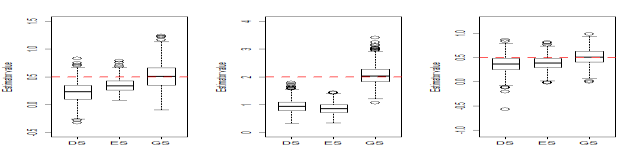


Fig. S4. Each boxplot summarizes the values of the parameter estimates (row 1 is for β, row 2 is for *κ*_1_, and row 3 is for *κ*_2_) that were obtaine with the empirical SSF (ES), the doughnut SSF (DS), and the gold standard (GS) methods from 500 simulations under the “discrete choice” kernel. The horizontal dashed line indicates the true parameter value.
