# Supplementary material for: Equivalence between Step Selection Functions and Biased Correlated Random Walks for Statistical Inference on Animal Movement
Source: PLoS One. 2015 Apr 21;10(4):e0122947. doi: 10.1371/journal.pone.0122947 (PMC4405542; doi:10.1371/journal.pone.0122947)
Supplement: S2 Appendix — Estimation of the standard errors of β^1 and β^2 using the variance covariance matrix of the κ^’s. (DOCX) [file pone.0122947.s002.docx]

**Equivalence between step selection functions and biased correlated random walks for statistical inference on animal movement. *PLoS ONE.***

S2 Appendix. *Estimation of the standard errors*

Let $\hat{V}$ be the 3×3 variance-covariance matrix of the estimators of the three κ coefficients. One has $\hat{\beta}_{1}=\hat{\kappa}_{2}/\hat{\kappa}_{1}$ and $\hat{\beta}_{2}=\hat{\kappa}_{3}/\hat{\kappa}_{1}$. Thus, the matrix of partial derivatives of $(\hat{\beta}_{1},\hat{\beta}_{2})$ with respect to $(\hat{\kappa}_{1},\hat{\kappa}_{2},\hat{\kappa}_{3})$ is

$\hat{A}=\left(\begin{matrix} -\hat{\kappa}_{2}/\hat{\kappa}_{1}^{2} & -\hat{\kappa}_{3}/\hat{\kappa}_{1}^{2} \\ 1/\hat{\kappa}_{1} & 0 \\ 0 & 1/\hat{\kappa}_{1} \end{matrix}\right)$ .

The variance-covariance matrix of $(\hat{\beta}_{1},\hat{\beta}_{2})$that was obtained with the multivariate delta method is the 2×2 matrix $\hat{A}^{T}\hat{V}\hat{A}$, where “T” denotes a matrix transpose, and the standard errors of $\hat{\beta}_{1}$ and $\hat{\beta}_{2}$ are the square roots of the diagonal elements of this 2×2 matrix.
